# Supplementary figures and images for: Encoder-decoder convolutional neural network for simple CT segmentation of COVID-19 infected lungs
Source: PeerJ Comput Sci. 2024 Jul 23;10:e2178. doi: 10.7717/peerj-cs.2178 (PMC11323195; doi:10.7717/peerj-cs.2178)

monitor training

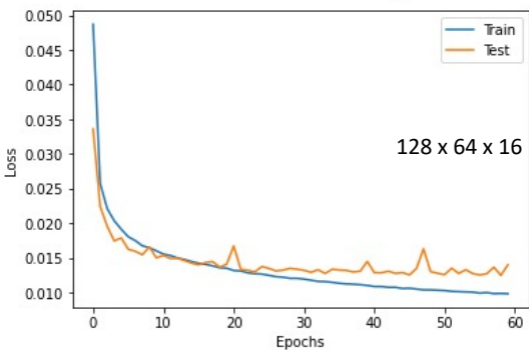

monitor training

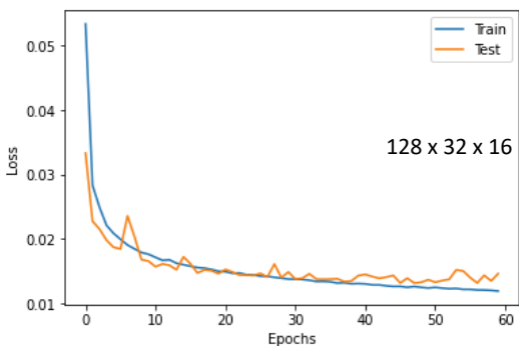

monitor training

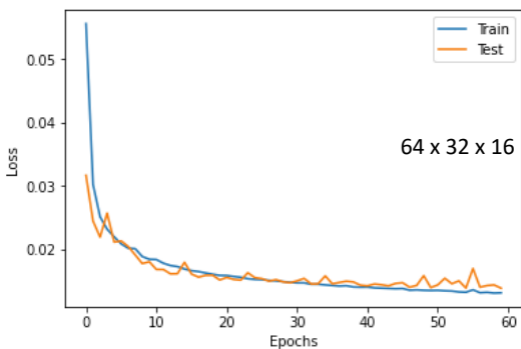

Supplement: Supplemental Information 1 — Selecting the best filter sizes for the overall shape of our ED-CNN model. [file peerj-cs-10-2178-s001.pdf]

monitor training

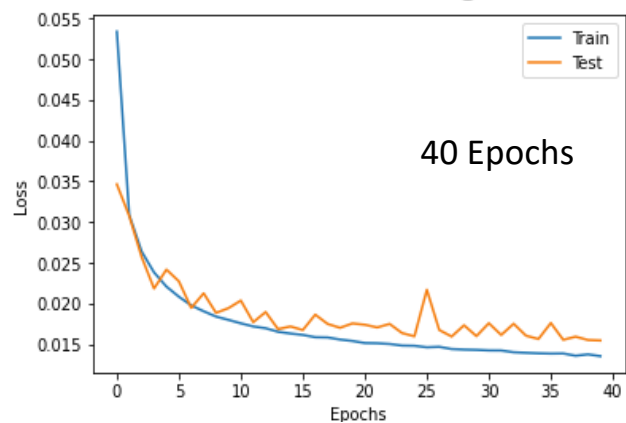

monitor training

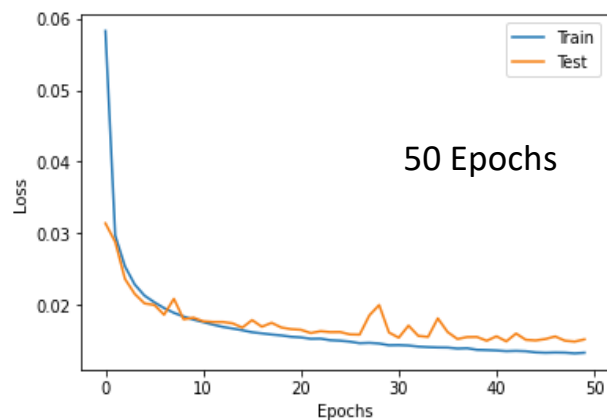

monitor training

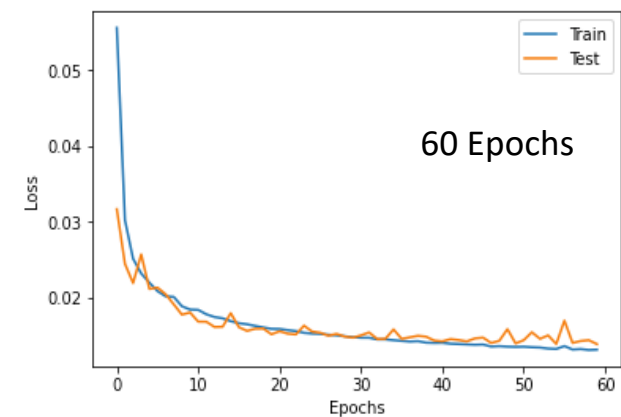

monitor training

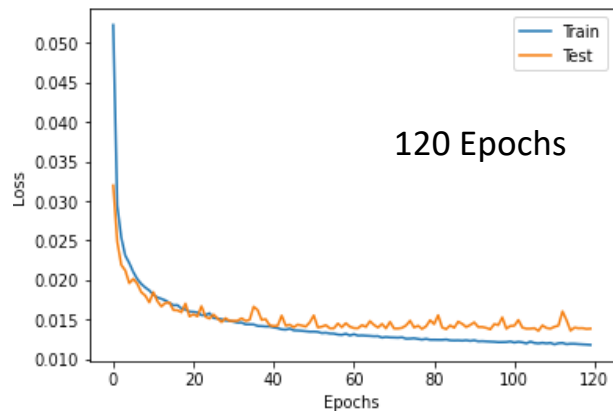

monitor training

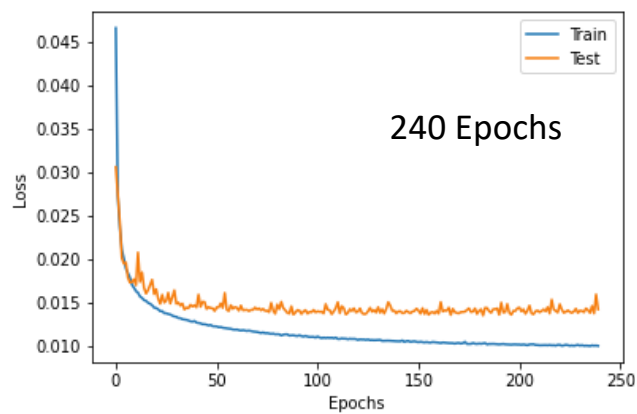

Supplement: Supplemental Information 3 — Testing Epoch values of 40, 50, 60, 120 and 240 to ensure the model isn’t over-fitting. [file peerj-cs-10-2178-s003.pdf]
