# Supplementary material for: Encoder-decoder convolutional neural network for simple CT segmentation of COVID-19 infected lungs
Source: PeerJ Comput Sci. 2024 Jul 23;10:e2178. doi: 10.7717/peerj-cs.2178 (PMC11323195; doi:10.7717/peerj-cs.2178)
Supplement: Supplemental Information 2 — This is used to increase the size of the training dataset from 1,475 initial images to 5,900 images after a series of flipping up/down and left/right to form a new larger training dataset. [file peerj-cs-10-2178-s002.pdf]

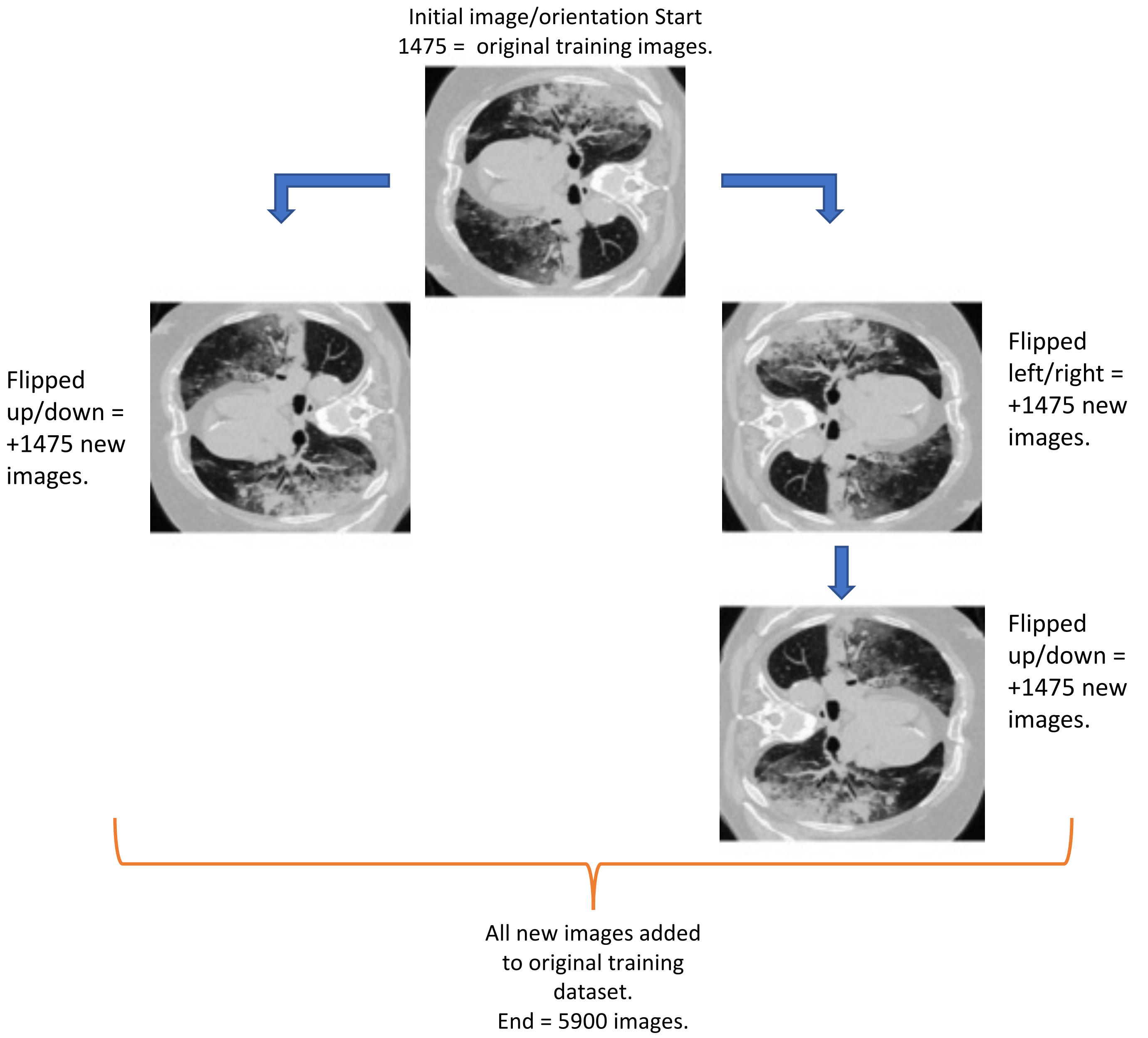
Initial image/orientation Start
1475 = original training images.
Flipped up/down = +1475 new images.
Flipped left/right = +1475 new images.
Flipped up/down = +1475 new images.
All new images added to original training dataset.
End = 5900 images.
