# Supplementary material for: Encoder-decoder convolutional neural network for simple CT segmentation of COVID-19 infected lungs
Source: PeerJ Comput Sci. 2024 Jul 23;10:e2178. doi: 10.7717/peerj-cs.2178 (PMC11323195; doi:10.7717/peerj-cs.2178)
Supplement: Supplemental Information 4 [file peerj-cs-10-2178-s004.pdf]

Supplementary Information for 'Encoder-Decoder Convolutional Neural Network for Simple CT Segmentation of COVID-19 Infected Lungs'

Kiri S. Newson[1], David M. Benoit[2], and Andrew W. Beavis[3,4,5]

[1]**Department of Physics and Mathematics, University of Hull, HU6 7RX, United Kingdom**
[2]**E. A. Milne Centre for Astrophysics, Department of Physics and Mathematics, University of Hull, HU6 7RX, United Kingdom**
[3]**Medical Physics Department, Queen's Centre for Oncology, Hull University Teaching Hospitals NHS Trust, Cottingham, HU16 5JQ, United Kingdom**
[4]**Medical Physics and Biomedical Engineering, University College London, London, WC1E 6BT, United Kingdom**
[5]**Hull York Medical School, University of Hull, Hull, HU6 7RX, United Kingdom**

Corresponding author:
Kiri S. Newson[1]

Email address: k.s.newson-2016@hull.ac.uk

ABSTRACT

This document contains supplementary information for the paper "Encoder-Decoder Convolutional Neural Network for Simple CT Segmentation of COVID-19 Infected Lungs" by Newson et al.

CODE AVAILABILITY

A copy of our Code for ED-CNN can be found at `https://github.com/Kiri-Newson/ED-CNN/blob/main/GitHub_version_ED_CNN_Model.ipynb` ///(DOI:10.5281/zenodo.8099854)

DATA AVAILABILITY

Data used throughout this work can be found online at `https://doi.org/10.5281/zenodo.3757476` via `http://medicalsegmentation.com/covid19/` (data from Jun et al. (2020)).

Repeats	Sm	Ea	MAE	Sen	Sp	Pc	F1	Acc
Run 1	0.8176	0.8644	0.0075	0.8342	0.9959	0.7284	0.7647	0.9939
Run 2	0.8017	0.8339	0.0065	0.6943	0.9984	0.8168	0.7335	0.9948
Run 3	0.8238	0.8568	0.0075	0.7913	0.9967	0.7999	0.7837	0.9934
Run 4	0.8338	0.8736	0.0077	0.8399	0.9955	0.7325	0.7708	0.9935
Run 5	0.8204	0.8736	0.0077	0.8147	0.9961	0.7665	0.7779	0.9934
Run 6	0.8234	0.8625	0.0078	0.8300	0.9959	0.7654	0.7806	0.9996
Run 7	0.8174	0.8670	0.0069	0.8062	0.9967	0.7656	0.7687	0.9946
Run 8	0.8194	0.8547	0.0071	0.7992	0.9972	0.7590	0.7623	0.9944
Run 9	0.8273	0.8628	0.0073	0.8355	0.9960	0.7410	0.7703	0.9939
Run 10	0.8166	0.8599	0.0079	0.8502	0.9955	0.6969	0.7463	0.9937

Table S1. Re-runs of our ED-CNN model - This is carried out with data randomisation each time - all computed with a different testing dataset with size of 369 images and different training set as a result of the randomisation when retraining the model.

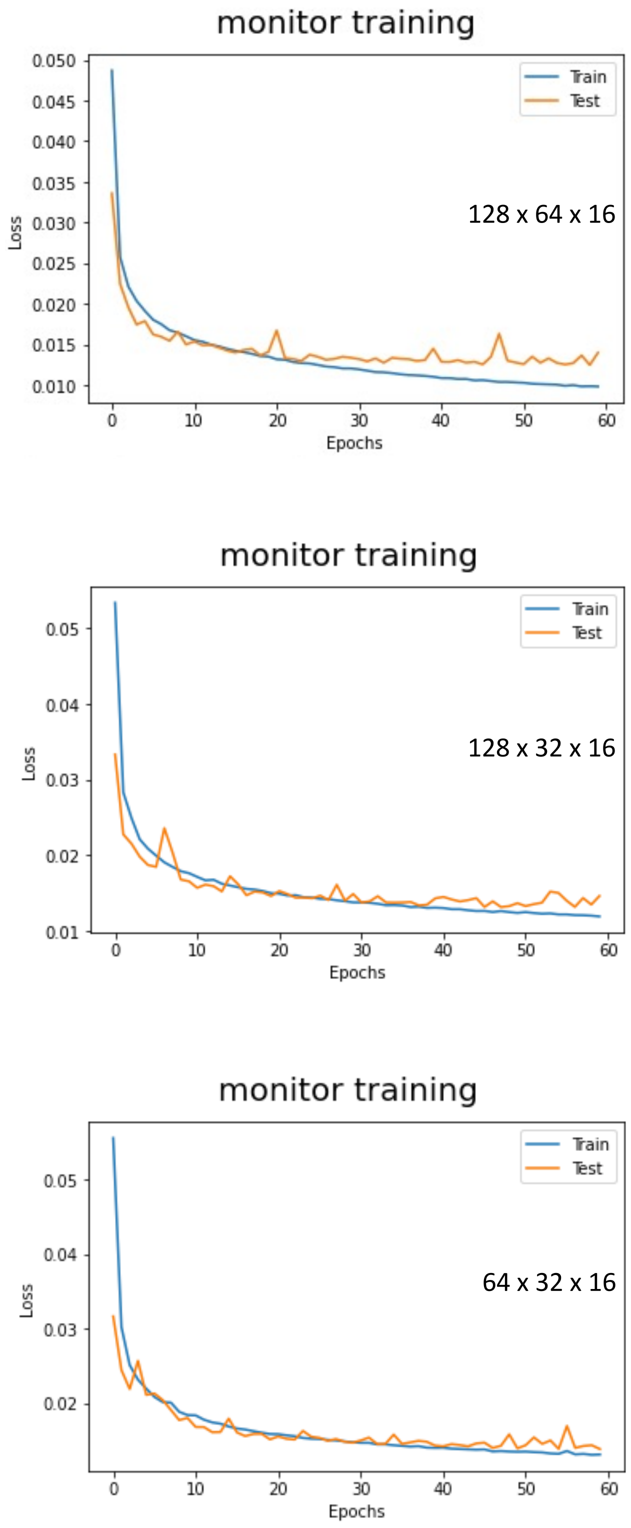

Figure S1. Investigating the best shape of the model based on training curves - Selecting the best filter sizes for the overall shape of our ED-CNN model

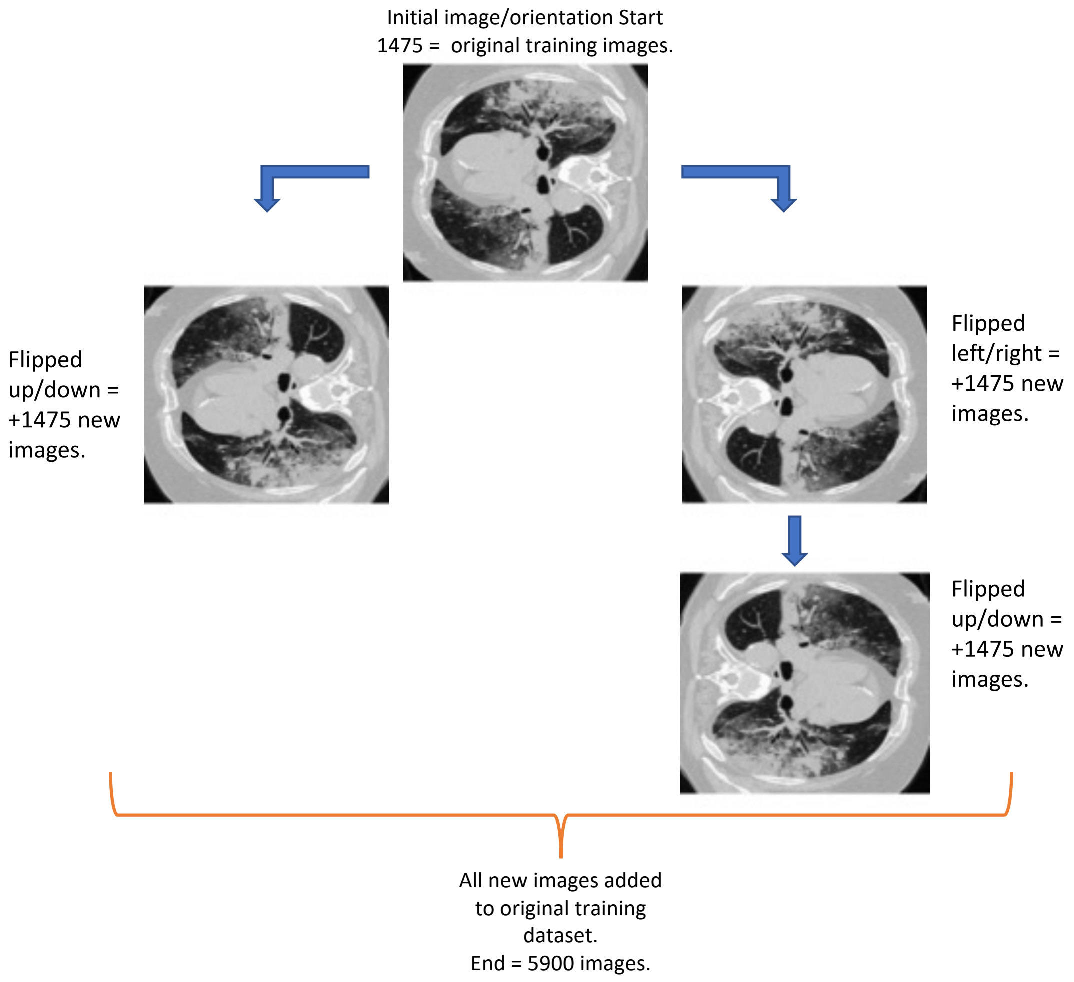

Figure S2. The data augmentation process - This is used to increase the size of the training dataset from 1,475 initial images to 5,900 images after a series of flipping up/down and left/right to form a new larger training dataset

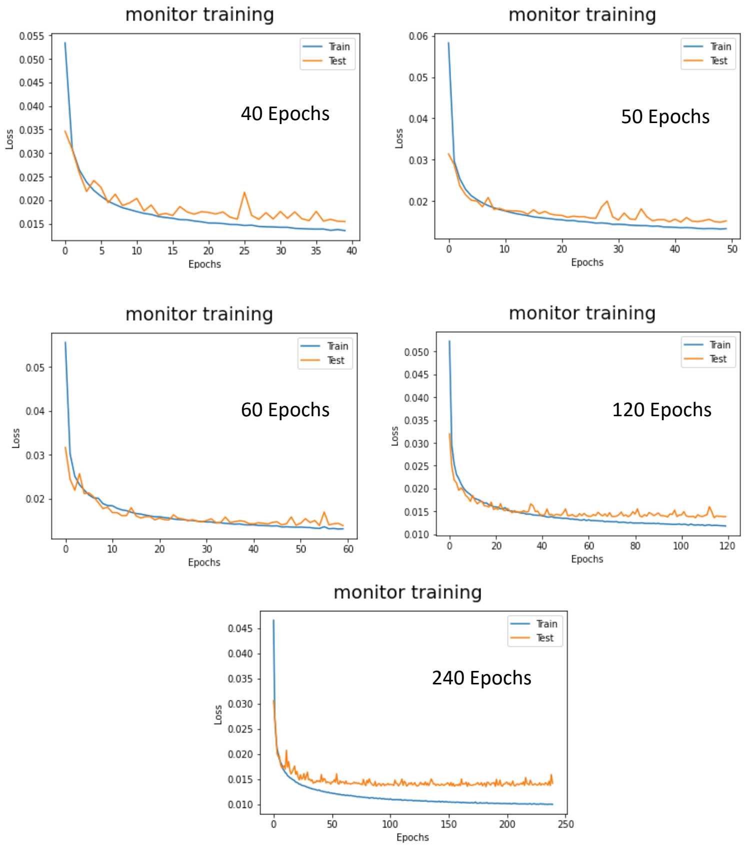

Figure S3. Testing a range of Epoch values - This is to ensure model isn't over-fitting.

REFERENCES

Jun, M., Cheng, G., Yixin, W., Xingle, A., Jiantao, G., Ziqi, Y., Minqing, Z., Xin, L., Xueyuan, D., Shucheng, C., Hao, W., Sen, M., Xiaoyu, Y., Ziwei, N., Chen, L., Lu, T., Yuntao, Z., Qiongjie, Z., Guoqiang, D., and Jian, H. (2020). COVID-19 CT Lung and Infection Segmentation Dataset.
